# A comparison of the efficacy of metronidazole vaginal gel and Myrtus (*Myrtus communis*) extract combination and metronidazole vaginal gel alone in the treatment of recurrent bacterial vaginosis

**Published:** 2017

**Authors:** Mansoureh Masoudi, Mahmoud Rafieian Kopaei, Sepideh Miraj

**Affiliations:** Medical Plants Research Center, Shahrekord University of Medical Sciences, Shahrekord, Iran

**Keywords:** Myrtus communis, Metronidazole, Bacterial vaginosis

## Abstract

**Objective:**

Due to the high incidence of bacterial vaginosis (BV) and its resistance to chemical medications and considering the anti-bacterial and anti-fungal effects of *Myrtus communis, *the present study aimed to compare the therapeutic effects of the vaginal gel of *M. communis *2% (in metronidazole base) with metronidazole vaginal gel 0.75% alone on BV.

**Materials and Methods:**

This research was a randomized controlled clinical trial conducted on 80 women of 18-40 years old with BV. Patients were divided into two groups of 40 women. Diagnostic criteria were Amsel's criteria and Gram staining. The first group received vaginal gel of metronidazole plus *M. communis *2% and the second group received metronidazole vaginal gel alone for five consecutive nights. Therapeutic effects and Amsel’s criteria were assessed after one week. Finally, the data were analyzed by SPSS 16 using *t-test* and Chi square tests.

**Results:**

There was a significant difference in the therapeutic response between the two groups. The results demonstrated that the combination of metronidazole and *M. communis* had a higher efficiency (p<0.05). The patients receiving *M. communis* in metronidazole gel base did not experience any recurrent BV, but 30% of patients taking metronidazole alone faced recurrent BV after three weeks of follow up.

**Conclusion:**

Findings of the study suggested that adding *M. communis* extract to metronidazole increases the efficiency of BV treatment.

## Introduction

Bacterial vaginosis (BV) which is the most prevalent form of vaginitis among women of reproductive age, is a revolution in bacterial flora of the vagina that leads to a decrease in native vaginal bacteria and overgrowth of anaerobic bacteria (Danforth and Gibbs, 2008 ▶). Since vaginitis causes problems for women, they seek for treatment (Farage et al., 2008 ▶). The most common infectious types of vaginitis are bacterial vaginosis, candidiasis, and trichomoniasis (Mashburn, 2012 ▶). The etiology of BV is not entirely known; however, researchers believe that BV is defined by a loss of the native lactobacilli producing hydrogen peroxide, and an overgrowth of anaerobic bacteria such as *Gardenela vaginalis*, *bactrioid species*, mobiluncus species and *Mycoplasma* (Danforth and Gibbs, 2008 ▶ ;Novak and Berek, 2007 ▶; Reichman et al., 2009 ▶; Allsworth, 2010 ▶). Vaginal lactobacilli acidify normal vagina environment, and inhibit the overgrowth of other vaginal bacteria, especially anaerobic bacteria (Ness et al., 2004 ▶; Riggs et al., 2007 ▶). 

Bacterial vaginosis has been related to various serious complications such as endometritis, pelvic inﬂammatory disease (PID), *Neisseria gonorrhoeae* and *Chlamydia trachomatis* infections, preterm delivery, chorioamnionitis, infections after gynecologic surgeries, spontaneous abortion, etc. (Danforth and Gibbs, 2008 ▶, Reichman et al. 2009 ▶, Klebanoff et al., 2010 ▶). Having multiple sex partners, inappropriate condom usage, smoking, and intrauterine contraceptive devices (IUDs) are the most important causes of BV (Farage et al., 2008 ▶; Schwebke et al., 2004 ▶; Koumans et al., 2007 ▶; Seyyedi F et al., 2016 ▶). Inversely, oral contraceptive pills prevent bacterial vaginosis (Klebanoff et al., 2010 ▶).

The most frequent symptom of BV is unpleasant vaginal discharge odor (Danforth and Gibbs, 2008 ▶, Klebanoff et al., 2010 ▶), which has negative outcomes on sexual and individual life of women (Leite et al., 2010). The clinical symptoms of BV include thin, grey-white, homogeneous and fishy-smelling discharge (Schwebke et al., 2004 ▶, Donders et al., 2014 ▶).

The major treatment of bacterial vaginosis is the usage of antibiotics (local or systemical). Antiseptics or probiotics can be also applied in treatment of BV. Metronidazole and clindamycin are two major antibiotics approved for treatment of BV. Furthermore, acidifying the vagina by acids such as lactic acid may enhance colonization of lactobacilli and prevent overgrowth of anaerobic microorganisms (Verstraelen et al., 2009 ▶; Löfmark et al., 2010 ▶).

Metronidazole has been successfully used for the treatment of anaerobic infections, septicemia, endocarditis, gynecologic infections, intra-abdominal infections, for more than 45 years (Brandt et al., 2008 ▶). Metronidazole is the key standard medication in treatment of BV with an effective activity against anaerobic bacteria and a low activity against lactobacilli. Metronidazole has considerable side effects as well. This antibiotic is metabolized in the liver. The concomitant use of metronidazole and anticoagulants such as warfarin causes prolongation of the prothrombin time. The most common complication of metronidazole is gastrointestinal problems including nausea, vomiting, metallic taste in the mouth and diarrhea. Furthermore, metronidazole causes other complications including drowsiness, dark-colored urine, dizziness, allergy, reduced transient neutropenia, candida infection, pancreatic inflammation, etc. Its infrequent serious complications such as peripheral neuropathy and convulsive seizures, described mainly by numbness or extremity paresthesia have been reported in patients taking prolonged metronidazole treatment (Brandt et al., 2008 ▶; Rafiean-Kopaei et al., 2012 ▶).

In recent years, the general population show a considerable tendency toward traditional treatments and medicinal herbs. Various research have evaluated antibacterial (Amirmohammadi et al., 2014 ▶; Sharafati-Chaleshtori and Rafieian-kopaei, 2014 ▶), antiparasitic (Shirzad et al., 2011 ▶; Bahmani and Rafieian-Kopaei, 2014 ▶) and anticancer (Ghassemi Dehkordi et al., 2003 ▶) effects of the medical plants. One of these herbs is *Myrtus communis* and numerous studies have reported its therapeutic properties

*M. communis *L, also known as Myrtus or Myrtle, belongs to the Myrtaceae family. This plant is an odorant, evergreen, perennial, erect shrub with maximum height of 5 m and a highly branched trunk. The young twigs of Myrtus contain glandular trichomes, simple, reciprocal and sharp leaves with a length of 5 cm. The leaves of Myrtus are mainly used in medicine. Some important compounds of the essential oil of Myrtus leaves are terpinyl acetate, geranyl valerate, hexyl butyrate and sabinene. Besides, the leaves contain tannin, flavonoid and vitamin C, without the existence of alkaloid and cardiac glycosides (Sobel, 2000 ▶). 

Traditional medicine believes that Mytrus has astringent, anti-diarrhea and antiseptic effects and strengthens hair growth. Mytrus was locally used in treatment of herpes, nasal mucosa inflammation. Extract of this herb can inhibit the growth of *Staphylococcus aureus*, *Pseudomonas aeruginosa* and *Escherichia coli* (Sobel, 2000 ▶).

Given the great prevalence of bacterial vaginosis, various side effects of chemical medications, resistance of microorganisms and due to the attitude of people toward herbal medicines , their fewer side effects and sometimes their higher efficacy, the present study aimed to compare the therapeutic effects of the vaginal gel of *M. communis *2% (in metronidazole base) and metronidazole vaginal gel 0.75% alone in women with BV referred to Women Clinics of Hajar Hospital, affiliated with Shahrekord University of Medical Sciences, Shahrekord, Iran.

## Materials and Methods

This study was a double-blind randomized controlled clinical trial. The trial was conducted on 80 married women aged 18-40 years with bacterial vaginosis referred to Women Clinics of Hajar Hospital, in 2014. 

The clinical identification of BV was performed by Amsel’s criteria and Gram staining. The criteria for patients were based on the existence of at least three out of the four Amsel’s criteria including white or gray homogeneous vaginal discharge, discharge pH > 4.5, clue cells more than 20 % in the vaginal wet smear, and positive “whiff” test (Simbar et al., 2008 ▶). Exclusion criteria were taking antibiotics during the past two weeks, postmenopausal, virginity and pregnancy, hysterectomy, bleeding, pelvic infections, women with diseases such as immune deficiency and diabetes, presence of sperm in smear and allergies to Myrtus.

Married women aged 18-40 years with vaginal discharge who referred to Women Clinics of Hajar Hospital, in 2014, were studied. After approving the study by the Ethics Committee of Shahrekord University of Medical Sciences and registering in the Iranian Registry of Clinical Trials with the IRCT code: 201411102085N13, the intent women were invited to contribute to this study, and informed about the aims and method of the study. All women signed an informed consent. In order to record the participants, they were given a questionnaire containing demographic characteristics, contraceptive method, number of pregnancies and medical history. Finally, eighty women were randomly registered, and randomly distributed to two groups of 40 individuals. Then, registered women were treated with either vaginal gel of *M. communis* 2% (Sobel and Ferris, 2006 ▶) (in metronidazole base) or metronidazole vaginal gel 0.75% alone for five consecutive nights.

This study was a double blind randomized clinical trial (neither statistical consultant nor physicians were aware of Drug Code).

The patients were evaluated one week after treatment completion for assessing therapeutic response. The vaginal discharge was cultured on HBT (Human Blood Tween) Bilayer Medium. HBT medium is a selective and differential medium used in the primary separation and probabilistic recognition of *Gardnerella vaginalis* from clinical specimens. Antibiogram of the isolated bacteria was prepared, and antibacterial effects of Myrtus extract were assessed in culture. In order to increase the precision of the study, the experiments were repeated three times.

Patients were not allowed to do vaginal washing, intercourse and use any vaginal medication during treatment. The remission and recurrence rates were followed 1 and 3 weeks after completion of treatment, in both groups. The lack of four Amsel's criteria or the presence of only one criterion of Amsel was indicator of the treatment’s success or failure, respectively (Rabiei et al., 2014 ▶). If the Amsel criteria were not present, it showed the success of treatment. If the Amsel criteria or criterion including; white or gray homogeneous vaginal discharge, pH> 4.5, clue cells more than 20 % in the vaginal wet smear, and positive “whiff” test//926), the treatment was unsuccessful.

Patients who did not show improvement seven days after treatment completion were excluded and referred for treatment with oral metronidazole.

**Extraction method**

After collecting the Myrtus leaves from Lordegan, confirmation of scientific name, and herbarium preparation, the percolation extraction method was performed by ethanol. Myrtus extract were added to metronidazole (vaginal gel of *M. communis* 2% in metronidazole base). Then, two groups of tubes were filled with metronidazole vaginal gel alone and metronidazole vaginal gel in combination with Myrtus. The shapes of tubes were identical and were not detectable by the physicians and patients.

**Determining antioxidant and anti-microbial activities of Myrtus extract**

The antioxidant activity of Myrtus extract was determined by 1-diphenyl-2-picrylhydrazyl (DPPH) assay (Sobel et al., 2006 ▶). The antimicrobial effects of Myrtus extract were estimated by determining its minimum inhibitory concentration (MIC). First, 20 mg of Myrtus extracts were dissolved in 1 ml physiological serum, and dilutions of 1.25, 2.5, 5, 10, and 20 mg/ml were prepared. Suspension of bacteria colonies available in specific medium of BHT with turbidity of 0.5 McFarland was added to dilutions and incubated at 37°C for 2-4 hr. Then, each dilution was inoculated into Methylene Blue (EMB) Agar medium and incubated at 37°C for 24 hr. Finally, the cultures were investigated based on growth or lack of growth of bacteria. 

**Statistical analysis**

Data were analyzed by SPSS software 16(Chicago), using frequency, mean, standard deviation, the chi-square test and independent t-test.

## Results

Here, 80 married women with mean age of 32.88±6.04 years old and age range of 19-40 years old participated in this study. Each group included 40 married women. Most participants in the study had a high school education (37.5%), and were housekeeper (85.8%). The natural method of contraception had higher frequency than other methods (34.2 %), and the groups were not significantly different (p =.76).

Also, 90.8% of patients had a history of vaginal infection, and 90.5% had received previous medical treatment. Moreover, 27.5% of women had a history of household treatment, and only 5% of them had a history of hospitalization because of infection. Mean of age, age at menarche, age at marriage, number of pregnancies, number of deliveries and abortions in two groups showed no significant difference. Besides, education and employment status are similar in the two groups (Table 1). History of infection, history of infection, hospitalization history because of vaginal infection, and household treatment of infection did not report any significant difference between two groups. In addition, there was no significant difference in clinical symptoms of BV before treatment, so the groups had similar clinical symptoms (Table 2). Symptoms of bacterial vaginosis in both groups after treatment were compared (Table 3).

**Table 1:** Demographic characteristics and confounding factor of the participants

		**Metronidazole Group** **Mean ± SD**	**Metronidazole and Myrtus Group** **Mean ± SD**	**p-value **
**Age (year)**		32.48±6.38	33.40±4.65	0.46
**Age at menarche (year)**		13.45±1.13	13.70±1.74	0.44
**Age of marriage (year)**		20.25±4.21	18.85±2.98	0.09
**Number of gestation**		2.18±1.31	2.50±1.24	0.26
**Number of delivery**		2.05±1.30	2.15±1.09	0.71
**Number of abortions**		0.25±0.54	0.35±0.66	0.46
**Employment status**	Employed	4 36	5 35	1
	Unemployed			
**Education**	Illiterate	2 19 19	2 19 19	0.50
	Below diploma			
	Diploma and upper			

a Data are presented as Mean ± SD.

**Table 2:** Comparison of symptoms of bacterial vaginosis in both groups before treatment

**symptoms**		**Metronidazole Group** **N %**	**Metronidazole and ** **Myrtus** ** Group** **N %**	**p-value**
**Irritation**	Yes	27 67.5 13 32.5	24 60 16 40	0.62
	No			
**Itching**	Yes	25 62.5 15 37.5	24 60 16 40	0.98
	No			
**Redness**	Yes	14 35 26 65	12 30 28 70	0.81
	No			
**Disparonia**	Yes	16 40 24 60	25 62.5 15 37.5	0.07
	No			
**Fever and Chills**	Yes	4 10 36 90	7 17.5 33 82.5	0.51
	No			
**Dysuria**	Yes	14 35 26 65	17 42.5 23 57.5	0.64
	No			
**Dysmenorrhea**	Yes	18 45 22 55	20 50 20 50	0.82
	No			

**Table 3:** Comparison of symptoms of bacterial vaginosis in groups after treatment

**symptoms**		**Metronidazole**		**communis L ** **Myrtus and Metronidazole**		**P value**
		**percent**	**frequency**	**frequency**	**percent**	
**Irritation**	yes	27.5	o.17	6	15	o.17
	no	72.5		34	85	
**Itching**	yes	37.5	0.48	12	30	0.48
	no	62.5		28	70	
**Redness**	yes	22.5	0.39	6	15	0.39
	no	77.5		34	85	
**Dyspareunia**	yes	10	0.40	2	5	0.40
	no	90		38	95	
**Fever and Chills**	yes	5	0.40	4	10	0.40
	no	95		36	90	
**Dysuria**	yes	15	0.26	9	22.5	0.26
	no	85		31	77.5	
**Dysmenorrhea**	yes	72/5	0.8	12	30	0.8
	no	72/5	29	28	70	

The two groups indicated no significant difference in frequency of remission using independent t-test (p <0.001) (Table 4). In addition, all patients were treated with metronidazole and Myrtus combination were satisfied with their remission, and examinations did not show reinfection 3 weeks after treatment completion. However, 12 patients (30%) in the metronidazole group encountered recurring infection.

**Table 4:** Comparison of recovery rate in groups

**Groups**	**Recovery**				
	**Without recovery**		**With recovery**		**p-value**
	**N**	**%**	**N**	**%**	
Metronidazole	17	42.5	23	5505	<0.001
Metronidazole and Myrtus	2	5	38	95	

## Discussion

We concluded that 2% metronidazole vaginal gel plus Myrtus extract, 5 grams once daily for five consecutive nights, has a better efficacy in treatment of BV than metronidazole vaginal gel alone, without any dangerous complications. The remission rate five days after the beginning of the treatment was better in group of Myrtus (in metronidazole base) than group of metronidazole vaginal gel alone. Besides, the recurrence ratio was 30% in the metronidazole group which is consistent with the consequences of study of Sobel and colleagues (25.5%) (Alipour et al., 2014 ▶).

Improvement was considered based on Amsel criteria, itching, burning, discharge, urinary frequency, disparonia, dysmenorrhea, age, occupation, education, and contraception method. In the readmission to track the effectiveness of treatment, presence or absence of Amsel criteria were considered as sign of remission (Sobel, 2000 ▶).

Laboratory investigation demonstrated that among the plates having different dilutions of Myrtus extract, bacteria grew only at the dilution of 1.25 mg/ml and no bacteria grew at dilutions 2.5-20 mg/ml. So, MIC of Myrtus extract was considered 2.5 mg/ml for bacterial vaginosis. Also, the amounts of phenols, flavonoids and flavonols of Myrtus extract were 91.08, 62.70 and 34.38 mg/g, respectively. Using DPPH method, antioxidant activity of Myrtus extract was determined 77.01μg/ml. This study is the first clinical trial assessing effect of metronidazole vaginal gel plus extract of Myrtus in the treatment of BV. Of course, several studies *in vitro* have demonstrated antimicrobial effects of Myrtus extract. 

Studies were performed on the chemical composition of Myrtus demonstrated that polyphenols, myrtucommulone, semimyrtucommulone, 1,8-cineole, α-pinene, myrtenyl acetate, limonene, linalool and α-terpinolene are among the most important ingredients of Myrtus that have biological activities. This herb has been traditionally used to treat bacterial and fungal infections (Yadegarinia et al., 2006; Houshmand et al., 2011 ▶).

Furthermore, the study of Houshmand et al showed that extract of Myrtus had anti-bacterial effects at different concentrations on aerobic and anaerobic bacteria (Al-Saimary et al., 2002 ▶). AL- Saimary believed that the anti-bacterial activity of Myrtus is due to the increase of oxygen free radicals and lipid peroxidation, which can damage the cell wall of microorganisms (Hosseinzadeh et al., 2011 ▶). Also, analgesic and anti-inflammatory effects of Myrtus have been demonstrated (Rossi et al., 2009 ▶). Therefore, therapeutic effects of Myrtus that have been reported by previous studies confirmed the results of the present study; so, Myrtus extract combined with metronidazole is more effective than metronidazole alone due to its anti-bacterial, anti-inflammatory, and analgesic properties. Patients taking this combination in the follow-up period of three weeks after the end of treatment did not experience recurrence of infection, while patients in the metronidazole group encountered lower remission and a recurrence rate of 30%.

Results of the study showed that the combination of metronidazole vaginal gel and Myrtus extract enhances the treatment efficiency of BV compared to metronidazole vaginal gel alone. We suggest more studies to be done for evaluating therapeutic effects of *M. communis* against other diseases. 
